# Reference Gene Selection for Quantitative Gene Expression Analysis in *Argynnis hyperbius*

**DOI:** 10.3390/insects16101008

**Published:** 2025-09-28

**Authors:** Hong-Juan Xin, Chen-Yang Liu, Feng Yan, Lu-Dan Wang, Huan-Huan Zhang, Chen-Hui Shen, Qing Zhai

**Affiliations:** 1 Henan International Laboratory for Green Pest Control, College of Plant Protection, Henan Agricultural University, Zhengzhou 450046, China; 2 Institute of Vegetable, Tibet Academy of Agricultural and Animal Husbandry Sciences, Lhasa 850032, China; 3 Key Laboratory of Agricultural Genetically Modified Organisms Traceability, Oil Crops Research Institute of Chinese Academy of Agricultural Science/Supervision and Test Center (Wuhan) for Plant Ecological Environment Safety, Ministry of Agriculture and Rural Affairs, Wuhan 430062, China

**Keywords:** *Argynnis hyperbius*, reference gene, RT-qPCR, selection, validation

## Abstract

*Argynnis hyperbius* (Lepidoptera: Nymphalidae)*,* as an important environmental indicator species, has shown a gradual decline in its species richness amid intensifying climate change and increasing environmental pressures. Screening for optimal reference genes is fundamental to studying their physiological and adaptive changes using RT-qPCR technology. In this study, 10 commonly used reference genes (*ACT*, *α-TUB*, *AK*, *GAPDH*, *EF1α*, *BTF3*, *RPS3*, *RPL10*, *RPL32*, and *RPL27*) were selected, and their expression stability under different developmental stages, genders, and temperature treatments was evaluated using the *RefFinder* website. The results indicated that selecting a pair of reference genes could achieve the most reliable normalization analysis under all experimental conditions. Specifically, *AK* and *EF1α* were the most stably expressed reference genes across different developmental stages; *ACT* and *RPL32* showed the most stable expression in different adult sexes; and *EF1α* and *RPL27* exhibited stable expression under different temperature treatments. Additionally, this study used *EF1α* and *RPL32*, the most stable reference genes from all results, to normalize and analyze the relative transcription levels of *HSP90* under different temperatures, revealing significant differences between the 4 °C group and both the 26 °C and 37 °C groups. The findings of this study will significantly improve the reliability of RT-qPCR detection and lay a foundation for in-depth research on the gene expression, physiology, and biochemical mechanisms of *A. hyperbius.*

## Simple Summary

*Argynnis hyperbius* (Lepidoptera: Nymphalidae) is widely distributed across China, utilizing *Viola philippica* (Malpighiales: Violaceae) as its primary host plant. Recent intensification of climate change and escalating environmental pressures have severely threatened species diversity. As ecologically significant indicator species, butterflies exhibit substantial variation in gene expression patterns across geographical populations and under divergent environmental conditions. Consequently, identifying appropriate reference genes is critical for accurate assessment of their physiological status and adaptive responses. This study systematically evaluated the expression stability of 10 candidate reference genes under diverse environmental stressors. Results demonstrate that *AK* and *EF1α* serve as optimal reference genes across developmental stages, *ACT* and *RPL32* show maximal expression stability between adult sexes, while *EF1α* and *RPL27* exhibit consistent expression under varying temperature regimes. These findings establish reliable reference genes for future gene expression studies in *A. hyperbius*.

## 1. Introduction

Real-time quantitative reverse transcription polymerase chain reaction (qRT-PCR) is widely applied for scientific and accurate quantitative analysis of gene expression, mRNA quantification, and related applications [1,2,3]. When utilizing qRT-PCR to measure gene expression levels in diverse organisms, it is essential to normalize the expression of multiple target genes against a stably expressed internal reference gene. This normalization process enhances the accuracy of quantitative results [4,5,6,7]. While reference genes are generally stable, no single gene can be universally applied to all scenarios [8,9,10,11]. The unstable expression of endogenous reference genes may result in erroneous gene expression data [12]. Therefore, it is essential to conduct validation under specific experimental conditions to identify the most suitable reference gene [9,13,14,15,16].

*Argynnis hyperbius* (Lepidoptera: Nymphalidae) is a complete metamorphosis and oligophagous butterfly species exhibiting distinct sexual dimorphism [17]. As an important pollinator of plants [18], butterflies play a dual ecological role in both maintaining food web dynamics and serving as a sensitive bioindicator for ecosystem health and stability [19,20,21]. Due to their high sensitivity to habitat alterations, butterflies have been widely recognized as a model organism for biodiversity studies [20,21], making significant contributions to ecosystem stability and diversity conservation [22]. Recent decades have witnessed a dramatic population decline of butterfly species worldwide. Gregory et al. calculated the average species index and designated specific indicators for each species in their analysis [23]. In response to this concerning trend, the European Environment Agency (2007) has advocated for establishing European butterfly indicators as a crucial biodiversity monitoring tool [24]. Currently, research on butterflies has mostly focused on the field of mitochondrial genomes [25,26], while the screening and application of reference genes have been more commonly seen in studies on moths [27]. In view of this, it has become particularly urgent to carry out the screening of reference genes in *A. hyperbiu*.

In this study, to determine suitable reference genes, ten commonly used candidates were selected based on the different developmental stages, temperature treatments, and sexes of *A. hyperbius*. Their stability was evaluated. The genes assessed were Actin (*ACT*), *α-tubulin* (*α-TUB*), basic transcription factor 3 (*BTF3*), arginine kinase (*AK*), glyceraldehyde-3-phosphate dehydrogenase (*GAPDH*), elongation factor 1α (*EF1α*), and the ribosomal protein genes (*RPS3, RPL10*, *RPL32*, and *RPL27*), which are frequently employed as reference genes for stability assessment [10,28,29]. Selecting the two to three most stable reference genes for normalization will facilitate future in-depth studies of *A. hyperbius* physiological processes, developmental mechanisms, and gene expression related to adaptation to environmental changes. Furthermore, the expression of *HSP90* in larvae under different temperature treatments was utilized to evaluate the normalization results. These findings provide a foundation for functional gene studies in *A. hyperbius*.

## 2. Materials and Methods

### 2.1. Insect Specimen Collection and Rearing

*A. hyperbius* specimens used in this study were collected in Pingdingshan City, Henan Province, China, in May 2024. The source population was initially collected within a 3 × 3 × 3 m net cage and reared under natural conditions. The larvae fed on *Viola philippica* (Malpighiales: Violaceae) and the adults sucked nectar.

### 2.2. Developmental Stage-Specific Sample Preparation

Larvae were fed on *Viola philippica*, while adults were provided with nectar sources. During rearing, specimens were sampled at all developmental stages: eggs, larvae (instars 1–6), pupae, and adults (both sexes). Twenty eggs, five larvae per instar group (instars 1–3), three larvae per instar group (instars 4–6), three pupae, three females, and three males were collected. All samples were placed in 2 mL centrifuge tubes and immediately stored at −80 °C.

### 2.3. Sample Collection Under Differential Temperature Regimes

Fifth and sixth instar larvae were transferred to 4 °C, 26 °C, and 37 °C (three larvae per temperature) for 6 h per treatment, totaling six experimental groups. Post-treatment, larvae were stored at −80 °C for subsequent RNA extraction.

### 2.4. Selection and Validation of Candidate Housekeeping Genes (HKGs)

Ten housekeeping genes (HKGs) were selected: Actin (*ACT*), *α-tubulin* (*α-TUB*), glyceraldehyde-3-phosphate dehydrogenase (*GAPDH*), elongation factor 1α (*EF1α*), ribosomal proteins *RPS3*, *RPL10*, *RPL27*, and *RPL32*, basic transcription factor 3 (*BTF3*), and arginine kinase (*AK*). Heat shock protein 90 (*HSP90*) was additionally selected. GenBank accession numbers are provided in Table 1.

Reference genes were amplified using primers listed in Table 1 via reverse transcription PCR. Amplification products were separated by 1.0% agarose gel electrophoresis and purified using a DNA purification kit. Purified DNA fragments were ligated into vectors, cloned, and bidirectionally sequenced. Obtained sequences were submitted to GenBank (accession numbers in Table 1).

### 2.5. RNA Extraction and cDNA Synthesis

Total RNA was isolated using TRIzol reagent (Invitrogen, Carlsbad, CA, USA) following the manufacturer’s protocol. RNA purity was assessed by measuring A260/A280 and A260/A230 ratios using a UV-1800 spectrophotometer (Shimadzu, Kyoto, Japan), with integrity immediately verified through 1.0% agarose gel electrophoresis. Reverse transcription was performed using gDNA wiper Mix enzyme with RNA templates to synthesize cDNA according to the manufacturer’s specifications. cDNA products were stored at −20 °C for subsequent use within 24 h.

### 2.6. Quantitative Real-Time PCR (qRT-PCR) Analysis

qRT-PCR primers (Table 2) were designed with Primer-BLAST software, and amplicons were validated by Sanger sequencing. Reactions were conducted on a CFX96 Real-Time System (Bio-Rad Laboratories, Hercules, CA, USA) using ChamQ Universal SYBR qPCR Master Mix (Vazyme Biotech Co., Ltd, Wuhan, China.). The 20 μL re*ACT*ion mixture contained: 10 μL 2× ChamQ Universal SYBR qPCR Master Mix, 1 μL cDNA template, 0.4 μL forward primer (10 μM), 0.4 μL reverse primer (10 μM), and 8.2 μL ddH_2_O. Each assay included reverse transcription-negative controls (without reverse transcriptase) and non-template controls. The thermal profile comprised: initial denaturation at 95 °C for 5 min; 35 cycles of 95 °C for 15 s, 50 °C for 15 s, and 72 °C for 2 min; followed by melting curve analysis (72 °C for 5 min, 12 °C for 2 min cycling). Reaction specificity was confirmed through melting curve analysis and gel electrophoresis using QuantStudio™ Design and Analysis software (v1.5.0). Amplification efficiency was calculated via 3-fold serial dilutions, with all reactions performed in triplicate.

### 2.7. Validation and Stability Assessment of Reference Genes

*HSP90* expression served to evaluate reference gene stability, amplified using primers:

Forward: 5′-TCTC*ACT*GACCCGTCAAAGC-3′

Reverse: 5′-GTAAGGGTGCCTTCGCTCTT-3′

Relative expression levels across temperatures were calculated in triplicate using the 2^−ΔΔCT^ method. Statistical analysis employed SPSS 26.0 (Chicago, IL, USA), with mean values (±SE) compared by the analysis of variance with Tukey–Kramer post hoc testing.

### 2.8. Data Processing and Statistical Analysis

Raw Ct values were obtained from QuantStudio 3™ Design and Analysis software (v1.5.0). Reference gene stability was evaluated using *geNorm*, *NormFinder*, and *BestKeeper* algorithms according to their respective manuals. Comprehensive rankings under each condition were generated by *RefFinder*.

## 3. Results

### 3.1. Screening and Characterization of Candidate HKGs

Ten candidate housekeeping genes (HKGs) were selected. The obtained GenBank accession numbers are provided in Table 1. All primer pairs exhibited amplification efficiencies between 92.75% and 105.94%, with slopes less than −3.0 and regression coefficients (R2) ranging from 0.993 to 0.999 (Table 2). These data confirm that primer performance meets standard qRT-PCR requirements [30].

### 3.2. Differential Expression Profiles of HKGs Across Experimental Conditions

Agarose gel electrophoresis of qRT-PCR products confirmed single amplicons of expected sizes for all ten genes, demonstrating their expression throughout the whole development, across temperature treatments, and in both sexes.

**Figure 1:**
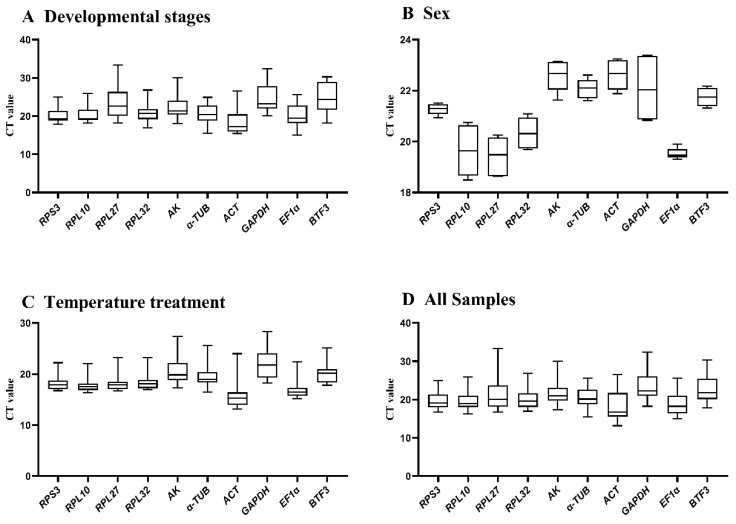
Expression stability of ten candidate reference genes in *A. hyperbius* evaluated by CT values. Boxplots display distributions across four experimental conditions: (**A**): developmental stages, (**B**): sexes, (**C**): temperature treatments, and (**D**): all the samples that have aggregated the first three groups of values together. Boxes represent interquartile ranges (25th–75th percentiles), with horizontal lines indicating medians. Data derived from three biological replicates. Abbreviations: *RPL10* (ribosomal protein L10), *RPL27* (ribosomal protein L27), *RPL32* (ribosomal protein L32), *RPS3* (ribosomal protein S3), *AK* (arginine kinase), *α-TUB* (*α-tubulin*), *ACT* (*Actin*), *GAPDH* (glyceraldehyde-3-phosphate dehydrogenase), *EF1α* (elongation factor 1α), *BTF3* (basic transcription factor 3). Abbreviations remain consistent in Figure 2, Figure 3, Figure 4 and Figure 5.

Expression stability was evaluated using threshold cycle (CT) values (Figure 1. During Developmental stages, *RPS3* and *RPL10* showed minimal variation, while *BTF3* and *GAPDH* exhibited substantial fluctuations (Figure 1A). Between adult sexes, *RPS3* and *EF1α* demonstrated low variation, whereas other genes displayed significant variability (Figure 1B). Under different temperature treatments, *AK* and *GAPDH* showed high variation, while the remaining eight genes maintained stable expression (Figure 1C). Collectively, *RPS3*, *RPL10*, *RPL32*, and *EF1α* exhibited minimal expression variation across conditions, whereas the other six HKGs showed substantial fluctuations.

### 3.3. Developmental Stage-Specific Stability of HKGs Expression

**Figure 2:**
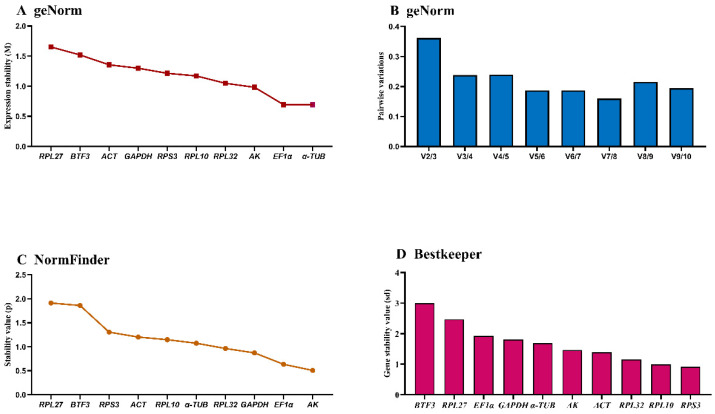
Expression stability of ten housekeeping genes during *A. hyperbius* development. Samples represent early/late larvae and pupae (days 1–2 per stage). The expression stability was ranked using three methods: (**A**) and (**B**): *geNorm*, (**C**): *NormFinder*, and (**D**): *BestKeeper*.

The *geNorm* algorithm evaluated candidate reference genes based on expression stability (M-value) and pairwise variation (Vn/Vn+1). *EF1α* and *α-TUB* demonstrated optimal stability during development (M-values: 0.5–1.5). *RPL27* and *BTF3* showed M-values > 1.5, while the remaining six genes exhibited intermediate stability (M-values ≈ 1) with comparable stability values (Figure 2A, Table 3). Pairwise variation analysis indicated V2/V3 > 0.15, supporting the requirement for three reference genes during developmental studies (Figure 2B).

*NormFinder* identified *α-TUB*, *EF1α*, *GAPDH*, and p as the most stable genes (stability values < 1.0), while *RPL27* and *BTF3* were the least stable (stability values ≈ 2.0) based on intra- and inter-group variation (Figure 2C, Table 3).

**Figure 3:**
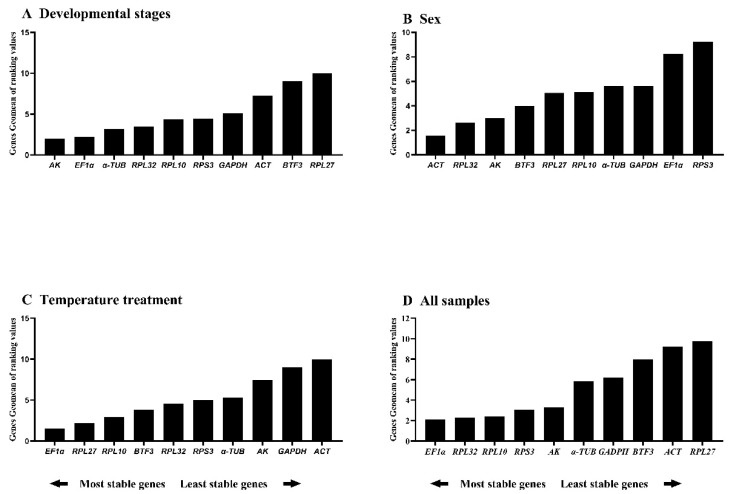
Comprehensive stability ranking of house-keeping genes across *A. hyperbius* experimental conditions. Figure displays distributions across four experimental conditions: (**A**): developmental stages, (**B**): sexes, (**C**): temperature treatments, and (**D**): all the samples that have aggregated the first three groups of values together. Rankings determined by *RefFinder* using the geomean method, where lower values indicate greater stability.

*BestKeeper* ranked stability as: *RPS3* > *RPL10* > *RPL32* > *ACT* > *AK* > *α-TUB* > *GAPDH* > *EF1α* > *RPL27* > *BTF3*. Only *RPS3* and *RPL10* showed standard deviation (SD) < 1; all others exceeded this threshold (Figure 2D, Table 3), indicating limited suitability as single reference genes. *RefFinder* integrated these analyses to generate a comprehensive stability ranking: *AK* > *EF1α* > *α-TUB* > *RPL32* > *RPL10* > *RPS3* > *GAPDH* > *ACT* > *BTF3* > *RPL27* (Figure 3A). Thus, *AK* and *EF1α* constitute the optimal reference gene combination for developmental stage expression studies.

### 3.4. Sex-Dimorphic Expression Stability in Adult HKGs

**Figure 4:**
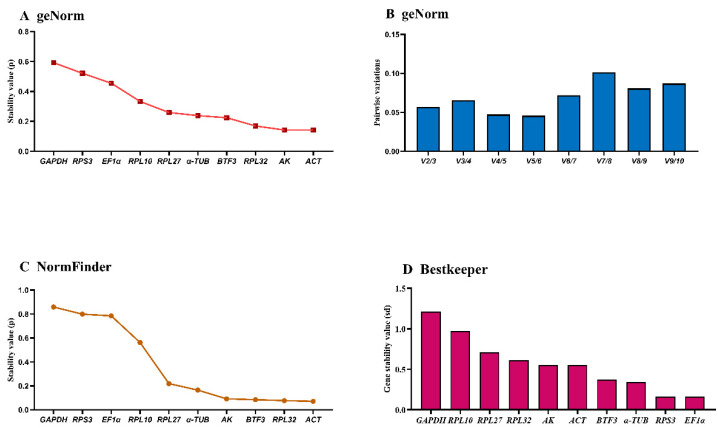
Expression stability of ten house-keeping genes in *A. hyperbius* adults. The expression stability was ranked using three methods: (**A**) and (**B**): *geNorm*, (**C**): *NormFinder*, and (**D**): *BestKeeper*.

The *geNorm* analysis revealed comparable stability across all ten reference genes (M-values < 0.6), with the stability ranking: *ACT* = *AK* > *RPL32* > *BTF3* > *α-TUB* > *RPL27* > *RPL10* > *EF1α* > *RPS3* > *GAPDH* (Figure 4A, Table 3). Pairwise variation (V2/V3-V9/V10 < 0.15) indicated all genes were suitable for expression normalization in both sexes (Figure 4B).

*NormFinder* ranked stability as: *ACT* > *RPL32* > *BTF3* > *AK* > *α-TUB* > *RPL27* > *RPL10* > *EF1α* > *RPS3* > *GAPDH,* with corresponding stability values: 0.859, 0.799, 0.785, 0.562, 0.219, 0.165, 0.092, 0.085, and 0.077. Genes with stability values < 0.4 (*RPL10*, *EF1α*, *RPS3*, *GAPDH*) demonstrated similar performance (Figure 4C, Table 3).

*BestKeeper* identified *EF1α* and *RPS3* as the most stable (SD = 0.16). *GAPDH* (SD > 1.0) showed low stability, while seven genes maintained SD < 1.0 (Figure 4D, Table 3).

*RefFinder* integration yielded the comprehensive ranking: *ACT* > *RPL32* > *AK* > *BTF3* > *RPL27* > *RPL10* > *α-TUB* > *GAPDH* > *EF1α* > *RPS3* (Figure 3B). Thus, *ACT* and *RPL32* are recommended as the optimal reference gene pair for expression studies in adult males and females.

### 3.5. Thermotolerance-Associated Stability of HKGs Under Temperatures Treatments

**Figure 5:**
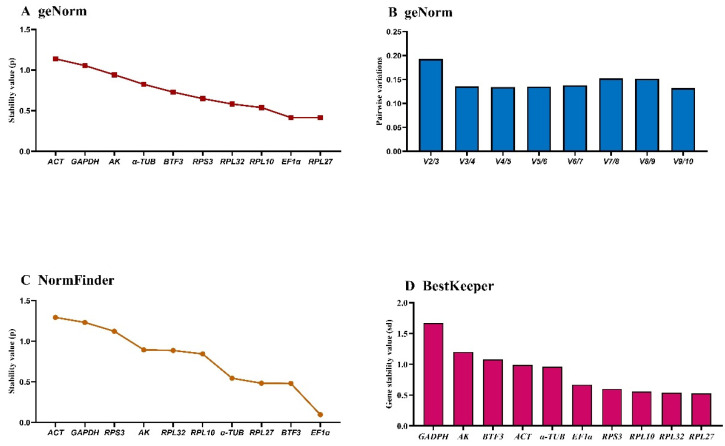
Expression stability of ten house-keeping genes in *A. hyperbius* under temperature treatments. Fourth- and fifth-instar larvae exposed to 4 °C, 26 °C, and 37 °C were analyzed. The expression stability was ranked using three methods: (**A**) and (**B**): *geNorm*, (**C**): *NormFinder*, and (**D**): *BestKeeper*.

The *geNorm* analysis ranked gene stability as: *RPL27* > *EF1α* > *RPL10* > *RPL32* > *RPS3* > *BTF3* > *α-TUB* > *AK* > *GAPDH* > *ACT* (Figure 5A, Table 3). *ACT* and *GAPDH* exhibited M-values > 1.0, while *RPL27* and *EF1α* showed optimal stability (M-values ≈ 0.5). The remaining six genes demonstrated intermediate stability (M-values = 0.5–1.0). Pairwise variation (V3/V4 < 0.15) indicated three reference genes are required for temperature treatments (Figure 5B).

The *NormFinder* identified *EF1α* > *BTF3* > *RPL27* > *α-TUB* > *RPL10* > *RPL32* > *AK* > *RPS3* > *GAPDH* > *ACT* as the stability ranking. *EF1α*, *BTF3*, *RPL27*, and *α-TUB* showed the lowest stability values, indicating comparable performance (Figure 5C).

*BestKeeper* analysis yielded: *RPL27* > *RPL32* > *RPL10* > *RPS3* > *EF1α* > *α-TUB* > *ACT* > *BTF3* > *AK* > *GAPDH* (Figure 5D, Table 3). *GAPDH*, *AK*, and *BTF3* (SD >1) demonstrated low stability, while the remaining seven genes (SD < 1) showed consistent performance. These genes exhibit stable expression and are validated as reference genes for qRT-PCR normalization when analyzing target gene expression levels.

*RefFinder* analysis revealed distinct stability rankings under specific versus combined experimental conditions. Under standardized conditions, the stability hierarchy was: *EF1α* > *RPL27* > *RPL10* > *BTF3* > *RPL32* > *RPS3* > *α-TUB* > *AK* > *GAPDH* > *ACT* (Figure 3C). However, when integrating data across three experimental conditions (developmental stages, temperature treatments, and sex differences), the composite stability ranking was: *EF1α* > *RPL32* > *RPL10* > *RPS3* > *AK* > *α-TUB* > *GAPDH* > *BTF3* > *ACT* > *RPL27* (Figure 3D). Notably, *EF1α* consistently ranked as the most stable reference gene in both analyses. Based on these comprehensive evaluations, we recommend the dual combination of *EF1α* and *RPL10* as optimal reference genes for qRT-PCR normalization across diverse experimental conditions in this model system.

### 3.6. Experimental Validation of Optimal Reference Genes for qRT-PCR Normalization

**Figure 6:**
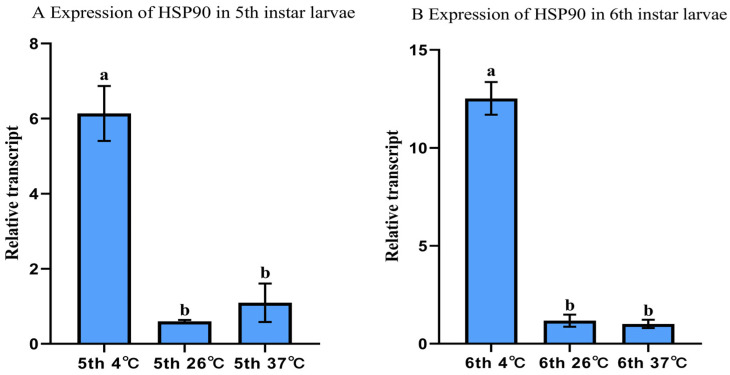
Relative *HSP90* expression in *A. hyperbius* larvae under temperature treatments ((**A**):fifth-instar; (**B**): sixth-instar). Fifth- and sixth-instar larvae exposed to 4 °C, 26 °C, and 37 °C were analyzed using qRT-PCR. Expression normalized to *EF1α* and *RPL32* and calculated via the 2^−ΔΔCT^ method, with 26 °C set as calibrator (value = 1). Columns represent means ± SE. Different letters denote significant differences (*p* < 0.05, ANOVA with Tukey–Kramer test).

To validate *EF1α* and *RPL32*, we analyzed *HSP90* expression in fifth and sixth-instar larvae subjected to different temperature treatments following normalization. *HSP90* exhibited the highest mRNA accumulation at 4 °C, intermediate at 26 °C, and lowest at 37 °C (Figure 6).

## 4. Discussion

Quantitative real-time PCR (qRT-PCR) represents the most widely adopted method for gene expression analysis [31,32]. Reliable reference genes are essential for ensuring quantitative accuracy. This study evaluated the expression stability of 10 housekeeping genes (HKGs) in *A. hyperbius*. *ACT*, *RPL*, *RPS*, *TUB*, *GAPDH*, and *EF1α* rank among the most commonly used reference genes [9,33,34].

Three experimental conditions were examined: developmental stages, temperature treatments, and sex differentiation—a standard approach in reference gene research. Significant expression variations were observed across treatments. *AK* and *EF1α* demonstrated optimal stability across developmental stages; *ACT* and *RPL32* exhibited maximal stability between sexes, while *EF1α* and *RPL27* maintained stability under temperature treatments. Discrepancies in stability rankings generated by *geNorm*, *NormFinder*, and *BestKeeper* likely reflect methodological differences in their statistical approaches [6,9,35,36]. *RefFinder* mitigates such discrepancies by calculating geometric means of algorithm-derived rankings [37,38].

*EF1α*, encoding a conserved cytoplasmic protein essential for protein synthesis [39,40], is widely employed as an insect reference gene [32,41,42]. Our findings confirm its stability across developmental stages, temperature treatments, and sexes—consistent with studies in *Drosophila* (Diptera: Drosophilidae) [3], *Cydia pomonella* (Lepidoptera: Tortricidae) [43], and *Phthorimaea operculella* (Lepidoptera: Gelechiidae) [14].

Arginine kinase (*AK*) is a phosphotransferase enzyme critical for cellular energy metabolism [44,45]. The arginine kinase gene showed high stability throughout *A. hyperbius* development, indicating its reference gene potential. In *Bombus terrestris* (Hymenoptera: Apidae), gene expression stability was highest in both labial gland and fat body tissues [46]. In *Dosophila uzukii* (Diptera: Drosophilidae), *AK* showed high stability as a reference gene [47]. Despite limited prior use [48], it exhibited remarkable stability during non-developmental stages in our study.

Actin (*ACT*), a crucial cytoskeletal protein, plays fundamental roles in various cellular processes, including muscle contraction and intracellular motility. Our systematic evaluation reveals distinct expression patterns across insect taxa. For example, consistent stability was observed in Lepidoptera, demonstrating stage-independent expression in *Phthorimaea operculella* (Lepidoptera: Gelechiidae) [3], *Plutella xylostella* (Lepidoptera: Plutellidae), and *Chilo suppressalis* (Lepidoptera: Pyralidae) [48]. Similar stability was documented in *Drosophila melanogaster* (Diptera: Drosophilidae) [3] and *Bemisia tabaci* (Hemiptera: Aleyrodidae) [49]. In contrast, expression variability characterizes certain Coleoptera species (Chrysomelidae and Henosepilachna) [11,50]. Futhermore, this study provides the first evidence of *ACT*’s sexual dimorphism-independent stability in adult *A. hyperbius*, establishing its utility as a novel reference gene for developmental and sex differentiation studies.

Ribosomal proteins (*RPs*), the primary structural constituents of ribosomes, assemble with four ribosomal RNAs to form functional subunits essential for cellular protein biosynthesis [51,52,53,54]. Extensive evidence supports RP-coding genes as exceptionally stable reference markers for insect gene expression quantification. Our systematic analysis identified condition-specific stability patterns: *RPL32* exhibited optimal stability across sex-dimorphic conditions, *RPL27* demonstrated temperature-resistant expression, while *RPL10* maintained constitutive stability under standard conditions. These findings correlate with established reports of: *RPL32* stability in *Phaedon brassicae* (Coleoptera: Chrysomelidae) under thermo-chemical stress [50]; *RPL19* invariance in *Colaphellus bowringi* (Coleoptera: Chrysomelidae) during photoperiodic adaptation [55]; *Helicoverpa armigera* (Lepidoptera: Noctuidae) *RPS15* and *RPL27* stability [56]; thermotolerant expression of *RPL10*, *RPL27,* and *RPS3* in *Mythimna loreyi* (Lepidoptera: Noctuidae) [57].

To validate the reliability of *EF1α* and *RPL32* as reference genes for qRT-PCR analysis in *Nymphalis xanthomelas* (Lepidoptera: Nymphalidae), we quantified *HSP90* transcript levels in 5th- and 6th-instar larvae under thermal stress conditions. The results demonstrated significantly elevated *HSP90* expression at 4 °C compared to 26 °C and 37 °C (Figure 6), consistent with the well-documented thermoregulatory function of heat shock proteins in stress response [58]. This pattern aligns with reported *HSP90* upregulation under cold stress in *Leptinotarsa decemlineata* (Coleoptera: Chrysomelidae) [59], *Nilaparvata lugens* (Hemiptera: Delphacidae) [60], and *Euzophera pyriella* (Lepidoptera: Pyralidae) [61]. The temperature-responsive differential expression of *HSP90* confirms the suitability of *EF1α* and *RPL32* as endogenous controls for gene expression normalization in this species.

## 5. Conclusions

Among the ten candidate reference genes evaluated in *Argynnis hyperbius*, *EF1α* and *RPL32* emerged as the most stable following comprehensive data integration. Specifically,

I. *AK* and *EF1α* demonstrated optimal stability across developmental stages
II. *ACT* and *RPL32* exhibited maximal expression stability between sexes
III. *EF1α* and *RPL27* maintained consistent expression under temperature treatments

This work establishes the first standardized qRT-PCR normalization framework for *A. hyperbius*, providing a robust foundation for future functional genomics investigations.

## Figures and Tables

**Table 1:** A list of primers used for RT-PCR of the genes.

Gene Name	Primer Sequences (5′ to 3′)	Amplicon Size (bp)	Accession Number
***RPS3***	Forward 5′-GA*ACT*TGCTGAGGATGGG-3 Reverse 5′-CAAGATAATCTAAGCCGTGAC-3	654	PV346079
***RPL10***	Forward 5′-TTCTTTTGCGAGGTGTCGGT-3 Reverse 5′-TCCTCCAAGAGTCGAGTGGT-3	667	PV346080
***RPL27***	Forward 5′-CTTGTCGACGGGTTAAGAGT-3 Reverse 5′-ACC*ACT*TGTTCTTTCCGCTCT-3	413	PV346081
***RPL32***	Forward 5′-TCAAAATGGCTATCAGACCTGT-3 Reverse 5′-TATTCGTTCTCCTGGCTGCG-3	409	PV346082
***AK***	Forward 5′-CCGACCATTCTGCACCTA-3 Reverse 5′-CAGCCAACATCCAAT*ACT*T-3	1202	PV346083
***α-TUB***	Forward 5′-ACGATTCCTTCAACACCTT-3 Reverse 5′-AGTATTCCTCAGCACCCTC	1215	PV346084
***ACT***	Forward 5′-GCGACGATGATGTTGCTGC-3 Reverse 5′-AGC*ACT*TGCGGTGGACGAT-3	1131	PV346085
***GAPDH***	Forward 5′-AATCTTATGACCCCTCTTTC-3 Reverse 5′-AT*ACT*CCAGCCGTGTCATT-3	999	PV346086
***EF1α***	Forward 5′-TGGTGGTATCGACAAACG-3 Reverse 5′-TTCGGTGAATGAAGTATCG-3	1294	PV346087
***BTF3***	Forward 5′-CGTGTCACGTGTAAAAATACCAGA-3 Reverse 5′-CA*ACT*TTCTTGTCCGCCGC-3	550	PV346088
***HSP90***	Forward 5′-ATTCTTCTGACGCTTTGG-3 Reverse 5′-TGCGAGAAGCATGAACCT-3	1887	PV346089

**Note:** *RPL10*, *RPL27*, *RPL32* and *RPS3*, ribosomal protein; *AK*, rginine kinase; *α-TUB*, α-tubulin; *ACT*, Actin; *GAPDH*, glyceraldehyde-3-phosphate dehydrogenase; *EF1α*, elongation factor 1α; *BTF3*, basic transcription factor 3; *HSP90*, heat shock protein 90.

**Table 2:** Primer sequences for 10 candidate reference genes in qRT-PCR analysis.

Gene	Primer Sequences (5′ to 3′)	Length (bp)	Slope	R2	Efficiency (%)
***RPS3***	F- 5′-GGCCTCATGATCC*ACT*CTGG-3	136	−3.320	0.993	100.08
	R- 5′-GGCCATTCTTGCCTTGTTGG-3				
***RPL10***	F- 5′-ATGGGGCTTCACCAAGTACG-3	85	−3.405	0.997	96.66
	R- 5′-GCGAACATTGCAGCCATCAT-3				
***RPL27***	F- 5′-AGCGCTCCAAAGTAAAGCCT-3	133	−3.462	0.999	94.46
	R- 5′-AGCTTCTTACGCTTAGCGGG-3v				
***RPL32***	F- 5′-GGTCCTCGTCCACAATGTCA-3	121	−3.486	0.996	93.59
	R- 5′-TTGCGCACGCTCAACAATAG-3				
***AK***	F- 5′-ATGCAAATGGGTGGTGACCT-3	130	−3.381	0.999	97.58
	R- 5′-CCAGGTTGGTAGGGCAGAAG-3				
***α-TUB***	F- 5′-GGTC*ACT*ACACCATCGGCAA-3	132	−3.480	0.999	93.82
	R- 5′-GAACCCTGATCCGGTACCAC-3				
***ACT***	F- 5′-ACGAAAGATTCCGTTGCCCT-3	146	−3.509	0.999	92.75
	R- 5′-AGACATGACAGTGTTGGCGT-3				
***GADPH***	F- 5′-GGCAAAGTTATCCCCGCTCT-3	105	−3.368	0.997	98.12
	R- 5′-TGGCTTACCAAGGCGTACAG-3				
***EF1α***	F- 5′-TGCGGCTATTGTCATCCTCC-3	130	−3.258	0.999	102.74
	R- 5′-GACAGCCTTGATGACACCGA-3				
***BTF3***	F- 5′-TCCCTGGCATCGAGGAAGTA-3	138	−3.367	0.999	98.15
	R- 5′-GGCCGAGCTG*ACT*CAAGATT-3				
***HSP90***	F- 5′-TCTC*ACT*GACCCGTCAAAGC-3 R- 5′-GTAAGGGTGCCTTCGCTCTT-3	81	−3.187	0.998	105.94

**Note:** *RPL10*, *RPL27*, *RPL32* and *RPS3*, ribosomal protein; *AK*, rginine kinase; *α-TUB*, α-tubulin; *ACT*, Actin; *GAPDH*, glyceraldehyde-3-phosphate dehydrogenase; *EF1α*, elongation factor 1α; *BTF3*, basic transcription factor 3; *HSP90*, heat shock protein 90.

**Table 3:** Expression stability of the candidate reference genes under different experimental conditions.

Conditions	CRGs *	*geNorm*		*NormFinder*		*BestKeeper*		ΔCt	
		Stability	Rank	Stability	Rank	Stability	Rank	Stability	Rank
**Developmental stages**	*RPS3*	1.212	6	1.304	8	0.92	1	1.68	8
	*RPL10*	1.171	5	1.147	6	0.99	2	1.58	6
	*RPL27*	1.652	10	1.912	10	2.47	9	2.18	10
	*RPL32*	1.049	4	0.964	4	1.16	3	1.5	3
	*AK*	0.982	3	0.508	1	1.46	5	1.34	1
	*α-TUB*	0.694	1	1.073	5	1.69	6	1.56	5
	*ACT*	1.355	8	1.202	7	1.39	4	1.67	7
	*GADPH*	1.299	7	0.873	3	1.81	7	1.51	4
	*EF1α*	*0.694*	1	0.635	2	1.92	8	1.37	2
	*BTF3*	*1.519*	9	1.86	9	2.99	10	2.13	9
**Sexes**	*RPS3*	0.521	9	0.799	9	0.16	1	0.84	9
	*RPL10*	0.333	7	0.562	7	0.97	9	0.65	7
	*RPL27*	0.259	6	0.219	6	0.71	8	0.48	5
	*RPL32*	0.169	3	0.077	2	0.61	7	0.44	2
	*AK*	0.142	1	0.092	4	0.55	5	0.46	3
	*α-TUB*	0.238	5	0.165	5	0.34	3	0.48	5
	*ACT*	0.142	1	0.071	1	0.55	5	0.42	1
	*GADPH*	0.592	10	0.859	10	1.21	10	0.88	10
	*EF1α*	0.455	8	0.785	8	0.16	1	0.82	8
	*BTF3*	0.224	4	0.085	3	0.37	4	0.46	3
**Temparature treatment**	*RPS3*	0.649	5	1.123	8	0.58	4	1.28	8
	*RPL10*	0.54	3	0.844	5	0.55	3	1.1	5
	*RPL27*	0.415	1	0.484	3	0.49	1	0.93	2
	*RPL32*	0.583	4	0.887	6	0.53	2	1.13	6
	*AK*	0.943	8	0.895	7	1.14	9	1.18	7
	*α-TUB*	0.825	7	0.545	4	0.86	6	1.03	4
	*ACT*	1.139	10	1.294	10	0.95	7	1.47	10
	*GADPH*	1.055	9	1.232	9	1.55	10	1.41	9
	*EF1α*	0.415	1	0.097	1	0.65	5	0.87	1
	*BTF3*	0.729	6	0.481	2	1.02	8	0.99	3

* Candidate reference genes.

## Data Availability

The original contributions presented in this study are included in the article. Further inquiries can be directed to the corresponding author(s).
